# Scaffolding in Health Sciences Education Programmes: An Integrative Review

**DOI:** 10.1007/s40670-022-01691-x

**Published:** 2022-12-07

**Authors:** Beloved Masava, Champion N. Nyoni, Yvonne Botma

**Affiliations:** School of Nursing, University of the Free State, Bloemfontein, South Africa

**Keywords:** Competence development, Fading, Health sciences programme, Integrative review, Modelling, Scaffolding, Student-centred learning

## Abstract

The complexity of health sciences programmes justifies scaffolding to support students in becoming competent health professionals. This article reports on an integrative review that aimed to describe the application of scaffolding in health sciences programmes. Twenty-nine sources, inclusive of theoretical and empirical studies, were reviewed. The sequencing of educational activities, the application of scaffolding tools or resources, frameworks for applying scaffolding, modelling, and fading represented the application of scaffolding in health sciences programmes. Awareness of the application of scaffolding in health sciences programmes could contribute to enhancing competence development among students when applied across all learning platforms.

## Introduction and Background

Students in health sciences programmes should be supported to adapt to the demands of learning complex skills and knowledge in constantly changing platforms, such as dynamic and evolving healthcare systems. Through scaffolding, educators support students’ learning by breaking down tasks and providing “just-in-time” strategies to enhance learning [1, 2]. Van De Pol et al. [3] define scaffolding as temporary support provided by an educator to aide students in completing a learning task that would prove difficult without such support. The concept has broadened to include scaffolding support that is presented as a designed or pre-planned structure applied at macro and meso-curriculum [4, 5], in addition to dynamic, contingent, adjustable support commonly described as instructional scaffolding, critical to enhance learning during educator – student(s) interactions [1, 6]. Scaffolding is an essential element of student-centred learning approaches [7].

Social constructivism underpins student-centred learning approaches [8], entailing interaction between the students and the educator in creating knowledge. The educator must be aware of students’ existing knowledge to design and employ appropriate learning activities that support the students past their zone of proximal development (ZPD), as conceptualised by Vygotsky [9]. Vygotsky [9] defined the ZPD as “the distance between students’ actual development as determined by independent problem-solving abilities and the students’ potential development as determined by problem solving with the assistance of a more capable peer or instructor”. The ZPD is influenced and informed by the curriculum, the educational programme, and the teaching and learning activities. Fading, a gradual withdrawal of educators’ support, promotes a seamless transition of students across the ZPD, ultimately enabling the transfer of the responsibility of learning to the student [3]. Modelling, knowledge application, scaffolding, and student achievement of mastery are necessary interventions to achieve internalisation and automatisation of knowledge [6, 10]. In health sciences education, problem solving integrates both cognitive strategies and execution of psychomotor skills.

There is global consensus in advocating for the adoption of student-centred approaches for health sciences education [11, 12]. The literature over the last decade revealed a general adoption of student-centred approaches to education with variable outcomes [13–17]. Adoption of student-centred approaches in health sciences education increases the need for scaffolding for competency development [13, 14, 18]. Additionally, the complexity of the disciplinary knowledge and skills in health sciences education necessitates scaffolding strategies.

Students in health sciences programmes develop competence in various contexts. Literature confirms that classrooms, simulation laboratories, community, and clinical environments are indispensable platforms for effective health sciences education [2, 7, 19–22]. These learning platforms are constantly changing in response to the dynamic and evolving healthcare systems. Students need support in adapting to these continuously changing platforms. Scaffolding as a support strategy has been reported to improve students’ comprehension of basic sciences knowledge [7], communication skills [23], evidence-based practice [18], academic literacy [24], clinical reasoning [2, 25], psychomotor [26], reflection [27, 28], and metacognitive skills [29] that are essential in adapting to dynamic learning platforms.

Literature demonstrates the value of scaffolding in higher education [2, 6, 30–32]. Student-centred health sciences programmes comprise complex, integrating psychomotor skills applied in various learning platforms. We argue that the complexity of health sciences programmes warrant various approaches to scaffolding. Although much has been written about scaffolding strategies in general, not enough is known about how it is applied in the health sciences. This article reports on an integrative review that describes how scaffolding is applied in health sciences programmes. Insight into the application of scaffolding within health sciences programmes may broaden knowledge about the various approaches to scaffolding and thus guide health sciences educators in applying appropriate scaffolding strategies to support student learning in this dynamic and constantly changing context.

## The Review

### Design

This integrative review answered the question “How is scaffolding applied in health sciences programmes?” The review followed the five integrative review stages described by Toronto and Remington [33] and Whittemore and Knafl [34], namely identification of a problem, the search and selecting the literature, data evaluation, data analysis and data presentation.

### Search Methods

An information specialist conducted a literature search in October 2020 using the following search string:

“Scaffold* OR Student support*” AND “Program* OR Module* OR Course* AND “Nurs* OR health science* OR health profession*”

### Search Outcomes and Selecting Literature

**Fig. 1:**
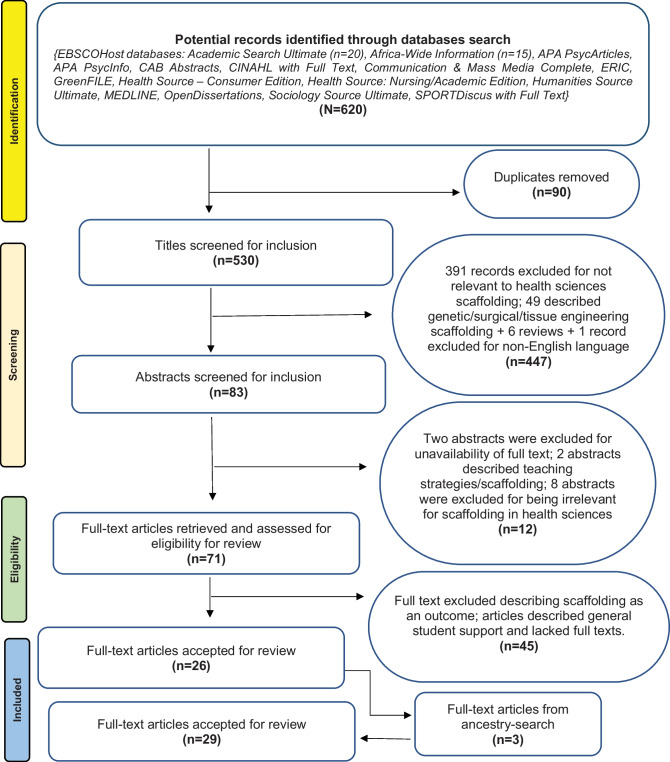
Adapted PRISMA flow diagram of selection of studies included in the review [35]

A total of 620 potential records containing titles and abstracts were sourced through the EBSCO host interface of a university library. Through automatic and manual deduplication, 90 records were removed. The three authors independently screened the remaining 530 records for relevance using an inclusion criterion (Fig. 1). Peer-reviewed articles and grey literature reporting on scaffolding in health sciences programmes since January 2010 were included. Literature in non-English languages was also excluded. The processes undertaken for identifying relevant publications, screening, and selecting the publications for review followed the Preferred Reporting Items for Systematic Reviews and Meta-Analyses (PRISMA) of 2020 [35], as shown in Fig. 1. The three authors did the screening, as they independently analysed text words contained in the titles and abstracts obtained from the initial search output. Authors held meetings to approve titles, abstracts or articles that met the inclusion criteria. An additional three articles were identified and included for review through an ancestry search of the reference list of accepted full-text articles. Finally, 29 sources were included in this integrative review (see Fig. 1 for more details regarding the selection and screening process).

### Article Appraisal

The Johns Hopkins University Research Evidence Appraisal Tools [36] were used to evaluate the methodological rigour of quantitative (*n* = 16) and qualitative (*n* = 1) studies included in this review. The remaining 12 articles were appraised using Johns Hopkins University non-research evidence appraisal checklist [36]. We did not eliminate articles secondary to the outcome of the appraisal, but instead, we sought to describe the extent of quality in the evidence on scaffolding obtained from the literature.

### Data Charting

A piloted author-generated data charting form was used to extract data from selected sources. The authors extracted characteristics of the sources, including authors names, type of publication, country of origin, study design, purpose of research, type of health sciences programme, methods applied, and the way scaffolding was applied (see Table 1).

**Table 1:** Study characteristics

**Citation**	**Type of publication**	**Country**	**Study purpose**	**Study type and design**	**HS programme – module**	**Setting**	**Theories used**	**Summary on how scaffolding was applied**
El Saadawi et al. [59]	Peer-reviewed article – *Advances in Health Sciences Education: Theory and Practice*	USA	To investigate the effect of metacognitive scaffolds and immediate feedback on students’ development of cognitive and metacognitive skills in pathology	Empirical – quantitative repeated measures design	Medicine – pathology	Computer-mediated	Self-regulated learning theory	Metacognitive scaffolds were embedded in SlideTutor computer program to support students’ diagnostic and reporting skills in terms of dermatopathology. The study demonstrated a positive effect of immediate feedback, colouring book, self-check summary, inspectable student mode and pseudo-dialogue using pre-stocked static questions in scaffolding the development of metacognitive and cognitive skills during teaching–learning of dermatopathology
Griffiths and Nicolls [61]	Peer-reviewed article – *Nurse Education in Practice*	UK	To evaluate an e-Support4U programme in scaffolding academic writing skills among students	Empirical – quantitative surveys	Nursing – (module unspecified)	Online learning platforms	Social constructivism	An evaluation of the effectiveness of the e-Support4U electronic-based learning platform, in enhancing students’ learning of academic writing skills during clinical placements. E-tivities were designed grounded in constructivism and based on Salmon’s 5-stage framework to structure and scaffold students’ academic writing learning. The 5 stages that guided structuring of e-tivities were access and motivation, online socialisation, information exchange, knowledge construction, and development
McKauge et al. [57]	Peer-reviewed article – *Journal of Pharmacy Practice*	Australia	To develop the scaffolding processes for clinical teaching–learning in an undergraduate pharmacy programme	Empirical – action research	Pharmacy – (module not mentioned)	Clinical settings	No theory mentioned	Used inputs from multiple stakeholders to develop graduated descriptors competency tool that outlined expected performances and pharmacy students’ experiential learning milestones. The tool scaffolded students in self-assessment and reflections on their learning milestones in relation to experiential learning. The tool also guided preceptors scaffolding strategies and structuring a dialogic feedback
Dawn et al. [37]	Peer-reviewed article – *American Journal of Pharmaceutical Education*	USA	To evaluate the effect of instructional scaffolding techniques and the clinical trial evaluation rubric on improving critical literature evaluation skills	Empirical –design not specified	Pharmacy programme – pharmacy informatics and research course	Classroom and skills laboratory	Scaffolding	Implementation of a “highly interactive, student-oriented and cognition-based approaches” to scaffold the development of competence in evaluating clinical literature among pharmacy students. Students are supported in working in individual, small groups and on class tasks. Scaffolding strategies supported students’ learning on text organisation, contextual information required, vocabulary evaluation, self-monitoring, regulation and metacognition
Colletti [44]	Grey literature –dissertation	USA	To describe the implementation of APLUS intervention to support authentic learning and critical thinking skills in allied health students	Empirical quantitative quasi-experimental	Respiratory care programmes – (module unspecified)	Online (virtual simulation)	Social constructivism	Applied APLUS (activate, plan, learn, use and show) to scaffold students in developing critical thinking skills. Students were tasked to manage authentic learning tasks whilst receiving prompt scaffolding
Cornelius [48]	Grey literature –dissertation	USA	To investigate the effect of visual thinking in improving critical thinking and sensitivity to physical examination competency	Empirical –participatory action research	Associate nursing programme – physical assessment	Classroom and clinical settings	Social constructivism, situated learning theory	The scaffolded design of physical assessment module teaching–learning activities to intertwine classroom and clinical learning opportunities. Scaffolding activities were reading of related material, educator modelling, using a visual thinking model, students practicing and developing nursing plans to manage the cases
Gormley et al. [55]	Peer-reviewed article – *Nurse Educator*	USA	To describe distributive scaffolding strategies to maximise student learning and motivation	Theoretical	Distance learning	Classroom and online learning platforms	Socio-cultural development and motivational theories	Presents general and non-specific online and traditional classroom-distributed scaffolding techniques. The techniques are instructing, questioning, modelling and feedback. Instructional strategies under these techniques are simulation, demonstrations, videos, audio feedback, concept mapping, talking aloud, exemplars, workbooks and guided notes
Peinhardt and Hagler [56]	Peer-reviewed article – *Journal of Nursing Education*	USA	To describe the effect of peer coaching as an intervention to support students’ writing abilities	Empirical – design not specified	Nursing –complex care theory course	Classroom	No theory mentioned	Used a structured response tool and process to guide the peer-coaching intervention to support students’ writing skills and application of evidence-based practice. The scaffolded assignments encouraged students’ interactions as they peer-reviewed draft assignments, evaluated content relevance and identified areas for revision
Stark [53]	Grey literature –dissertation	USA	To evaluate an instructional scaffolding intervention	Empirical quantitative quasi-experimental	Nursing – module not specified	Classroom	Social constructivism	Used instructional scaffolding strategies of question prompts, modelling, thinking aloud, alternative perspectives to support students’ problem solving and metacognition abilities on ill-structured problems. The use of scaffolding reduced cognitive load during problem solving
Pretorius et al. [65]	Peer-reviewed article – *International Journal of Teaching and Learning in Higher Education*	Australia	To demonstrate the effective use of research skills development and constructive alignment theory in scaffolding a midwifery assessment task	Empirical –quantitative surveys	Bachelor of Nursing & Midwifery –supporting birthing women	Classroom	Constructivist theory	Used constructive alignment and Research Skills Development (RSD) framework to incorporate research and evidence-based practice in re-designing a midwifery assignment. Initially, students learned about research skills in RSD, guidelines on using evidence-based practice, and an interactive workshop where peers collaborated on how to answer the clinical question in the assignment. The RSD framework and facilitator guidance in the form of question prompts supported students’ learning of critical thinking and research skills
Lefroy et al. [67]	Peer-reviewed article – *Advances in Health Sciences Education: theory and practice*	UK	To develop strategies used as content scaffolds to improve specificity of feedback on consultation skills	Empirical _design not specified	Medicine_ Module not specified	Clinical settings	No theory mentioned	The authors developed a repository of specific content or strategies to scaffold and improve specificity of feedback on consultation skills. The Generic Consultation Skills (GeCoS) list allows the facilitator to draw automated feedback from ready-made strategies on scaffolding students with deficiencies in performance. Strategies also act as a standard or exemplar for students to base on during self-evaluation
Dukes [60]	Grey literature –dissertation	USA	To describe the use of a SIDNIE virtual platform in scaffolding simulation learning	Empirical -action research	Bachelor of Science in Nursing (BSN) – module not specified	Virtual simulations	Scaffolding	Used an electronic platform, SIDNIE to support student nurses learning of interviewing skills. Provided as a virtual platform, SIDNIE dynamically adapts scaffolding and customise feedback relative to the students’ performance. The system allows fading or removal of scaffolds to promote independent practise, automatisation and self-regulation
Leppink and Duvivier [45]	Peer-reviewed article – *Medical Teacher*	The Netherlands	To present instructional design tips using cognitive load theory to design a curriculum that facilitates learning from high instructional support on low complex low fidelity tasks to independent practice of high complex tasks in challenging environments	Theoretical	Medicine – module not specified	Not mentioned	Cognitive load theory	The article proposes scaffolded instructional design tips that use cognitive load theory. The scaffolded curriculum seek to move students from low-fidelity, low-complexity and high-supportive contexts to competent, independent performance in high-complexity and high-fidelity contexts. As students move from one level of fidelity and complexity to another, adequate support is paramount to decrease cognitive overload and frustrations
Skinner et al. [49]	Peer-reviewed article – *Journal of Learning Design*	Australia	To implement consistent and scaffolded participation of physiotherapy students in experiential learning using small group and student-centred approaches	Empirical – quantitative surveys	Undergraduate physiotherapy degree – (module unspecified)	Classroom and clinical settings	Experiential learning theory	Utilised student-centred and small-group approaches to implement consistent and scaffolded participation of students in experiential learning. Each week small groups of students participated in tutorials, lectures, practicals and plenary sessions to cover physiotherapy disciplinary content and communication skills. The content and skills covered were scaffolded horizontally within a year and vertically across the programme to enhance competence development among students
Fletcher and Meyer [58]	Peer-reviewed article – *Journal of Professional Nursing*	USA	To describe how a playbook was scaffolded to build the students’ competencies over the course of the programme	Theoretical	Nursing programme – clinical course	Online learning platforms and clinical settings	Transformative learning theory	Focused Learning Assignments (FLAs) collected in the form of a playbook exposed students to structured, systematic and transformative learning opportunities throughout the nursing programme. The playbook was structured into various competencies including, safe quality education, systems-based practice and professionalism which build on each other in increasing complexity. The playbook covered content across semesters, building competencies across the programme curriculum
Lauerer et al. [38]	Peer-reviewed article – *Nurse Education Today*	USA	To design a scaffolded curriculum of behavioural health concepts	Theoretical	Doctor of Nursing Practice (DNP) primary care programme – clinical course	Classroom and virtual learning platforms	Constructivist learning theory	A scaffolded curriculum design was adopted to integrate behavioural healthcare content into a primary care module. The module was delivered through an online Problem Based Learning (PBL) of primary care nursing alternated with learning intensives. The scaffolded design encouraged students to build upon previous knowledge
Frantz and Rhoda [39]	Peer-reviewed article – *Journal of Interprofessional Care*	South Africa	To present lessons learnt when implementing a scaffolding approach to teaching activities within an IPE curriculum	Theoretical	Inter-Professional programme – IPE module	Not mentioned	No theory mentioned	An Inter-Professional Education (IPE) implementation plan was designed incorporating scaffolding principles. The design covered the six collaborative competencies spread over three years. The scaffolding design promoted structuring and sequencing of IPE content from simple foundational tasks through intermediate tasks until students could independently manage real cases requiring complex solutions under authentic conditions
Luo and Kalman [47]	Peer-reviewed article – *Nursing Forum*	USA	To describe the design and development of a 12-step technology training protocol to support learning of new software by nurses	Empirical –qualitative	Nursing – module not specified	Classroom	Experiential learning theory	A scaffolding strategy to facilitate learning new software among nurses was presented as a series of steps that followed the experiential learning theory. Nurses used previous knowledge to navigate the application of procedural workflow on new software. Question prompts and guiding instructions were used when stuck to easy the process and reduce students’ cognitive load
Duff et al. [42]	Peer-reviewed article – *Nurse Education in Practice*	Australia	To describe how an innovative oral health education module was designed to incorporate scaffolding principles	Theoretical	Bachelor of Midwifery – oral health education module	Classroom and online learning platforms	No theory mentioned	Innovative components of an oral health module were integrated into a midwifery curriculum to build a hierarchy of knowledge construction from basic concepts to application. The module design followed a low- to high-fidelity trajectory where content on foundational knowledge was covered first before students were assigned scenarios to assess, diagnose, manage and refer patients with oral pathologies
Braun et al. [62]	Peer-reviewed article – *Medical Education*	Germany	To investigate the effect of representation scaffolding on the diagnostic efficiency of intermediate medical students	Empirical quantitative – Randomised controlled laboratory study	Medicine – internal medicine	Virtual simulations	No theory mentioned	Authors investigated how case representation scaffolds could improve diagnostic efficiency and accuracy among students using an electronic case simulation platform. Students worked with CASUS on four learning cases and four assessment cases. The intervention group, who received prompts for representation scaffold, demonstrated significant improvement in diagnostic efficiency
Herrington and Schneidereith [52]	Peer-reviewed article – *Nurse Educator*	USA	To design a scaffolded simulation-integrated nursing curriculum	Empirical – quantitative surveys	Nursing programmes – (module not specified)	Skills laboratory	No theory mentioned	Stakeholder inputs were sought to structure and sequence concept-based simulations in undergraduate nursing programmes following Benner's competency framework. Scaffolding allowed meaningful, coherent connections across the programme, curricular-enhancing integration of simulations in the programmes
Zook et al. [40]	Peer-reviewed article – *Nurse Educator*	USA	To describe the scaffolding design and implementation of an inter-professional education curriculum	Empirical –quantitative – prospective and non-experimental 1-group longitudinal study	Doctor of nursing practice, psychology, and speech-language pathology – (inter-professional education course [IPE])	Online and simulation platforms	No theory mentioned	A multi-semester IPE curriculum was reviewed to structure and sequence its content from simple to complex situations. Through online platforms, students began the module by learning foundational IPE and teamwork principles. Later, students worked on virtual simulations and team challenges to solve complex patient problems and create relevant care plans
Coombs [41]	Peer-reviewed article – *Nurse Education Today*	Australia	To describe a scaffolded design and process to facilitate learning in a nursing course	Theoretical	Bachelor of Nursing degree – determinants of health and health, promotion and illness prevention	Classroom and online learning platforms	Constructive alignment theory	Strategic scaffolding and constructive alignment were applied in design and as a process to support students’ learning from simple manageable tasks to complex task performances under progressively fading guidance. Through scaffolding, students integrated foundational knowledge on health determinants, population health needs assessment, interpreting, diagnosing to develop a health promotion programme to manage identified problems
Miller et al. [46]	Peer-reviewed article – *Nurse Education in Practice*	USA	To compare implementation of a scaffolded sequence of writing assignments (intervention) to typical writing assignments (comparison)	Empirical – quantitative quasi-experimental	Baccalaureate Nursing (BSN) – Evidence-based nursing practice and Effectiveness in complex health systems	Classroom and virtual learning platforms	Scaffolding	A single-semester writing assignment was designed using a scaffolding sequencing approach. The assignment required students to respond to an evidence-based practice question. Strategic sequencing, rubrics, peer and instructor feedback scaffolded students’ abilities dealing with simple tasks, simple focus assignments and complex tasks that required integration of diverse types of knowledge
Banerjee et al. [68]	Peer-reviewed article – *BMC Medical Education*	United Arab Emirates	To describe the use of 6-D approach in scaffolding students in learning of basic science concepts	Theoretical	Medicine – molecular biology and principles of genetics	Classroom	Social constructivism, humanist and experiential learning theories	A 6-D approach is presented to structure the learning of basic science concepts utilising mentored journal clubs. After didactic lectures on concept basics, groups of students participated in searching and distributing related literature; constructing knowledge, presenting, discussing and critically evaluating the evidence brought forward to class. Educator supports students in first three stages before fading in the last two
Theobald and Ramsbotham [43]	Peer-reviewed article – *Nurse Education in Practice*	Australia	To investigate if inquiry-based learning and using a clinical reasoning framework and accompanying scaffolds to support teaching and learning of clinical reasoning	Empirical – action research	Bachelor of Nursing course – (Nursing theory)	Classroom	Social constructionism	Used an inquiry-based learning approach, which embedded a clinical reasoning framework, questioning prompts, modelling and exemplars to teach clinical reasoning skills to students. The support enhanced students’ problem solving abilities on given cases and authentic tasks
Sasa [69]	Peer-reviewed article – *Teaching and Learning in Nursing*	USA	To describe how a structured writing assignment scaffolded students’ writing skills in clinical courses	Theoretical	Associate degree in nursing– (medical-surgical nursing course)	Clinical settings	No theory mentioned	The Nursing Care Paper (NCP) report was structured following the nursing process. The assignment was scaffolded and sequenced by tasking students to work initially on low-task weekly reflections that focused on a particular stage of the nursing process. The results of reflections culminated in creation of high-stake case assignments on the nursing care plan of a patient
Bingen et al. [50]	Peer-reviewed article – *Journal of Clinical Nursing*	Norway	To explore the use of flipped classrooms in supporting students’ mastery of physiology	Empirical – design-based	Bachelor of Nursing degree – anatomy and physiology	Classroom and online learning platforms	No theory mentioned	Used design-based research to design flipped classroom to support students’ mastery of physiology content. Flipped classroom activities were learner-centred, collaborative, used digital tools, and provided necessary foundational scaffolding for students’ mastery of physiology content
Chambers et al. [66]	Peer-reviewed article – *MedEdPORTAL, The AAMC Journal of Teaching and Learning Resources*	USA	To describe how an Inter-professional curriculum scaffolded graduates in learning complex procedural skills and inter-professional practice required for patient safety	Empirical –quantitative surveys	Inter-professional education programme – venepuncture and Peripheral Intravenous Catheter (PIV) insertion	Skills laboratory	No theory mentioned	The deliberate design of the inter-professional PIV module to follow the Sawyer’s “learn, see, practice, prove, do and maintain” framework. The framework fosters the development of medical students’ procedural knowledge on venepuncture and PIV insertion. The module guide broke the procedures into 4 phases: planning, preparation, insertion, and post-venepuncture care to further structure and strengthen scaffolding on learning of the skill

### Data Analysis

After charting the sources, data was presented in an Excel spreadsheet. Analysis was conducted per characteristic. Frequencies were calculated for categorical data. Inductive analysis was applied to narrative data as authors read through the extracted data on scaffolding applications in health sciences programmes, analysing content, observing common trends, and patterns shared. Furthermore, the outcomes of the inductive analysis were synthesised to create themes and subthemes in terms of the application of scaffolding across various learning platforms.

## Results

Data analysis and synthesis yielded results on characteristics of the studies and themes regarding the application of scaffolding in health sciences programmes.

### Study Characteristics

The majority of the sources were from the USA (*n* = 16), Australia (*n* = 6), and the UK (*n* = 2), while the Netherlands, Norway, Germany, South Africa, and the United Arab Emirates contributed one source each. Seventeen of the sources were studies on scaffolding in nursing and/or midwifery programmes, followed by medicine (*n* = 5), pharmacy (*n* = 2), allied health (*n* = 2), and inter-professional education (IPE) (*n* = 3). The majority of these sources reported the application of scaffolding in undergraduate programmes (*n* = 27). Of the 19 sources that reported on the specific modules where scaffolding was applied, the modules were in the hard sciences (*n* = 2), clinical skills (*n* = 9), in discipline-related knowledge (*n* = 6), and in academic writing, research, and evidence-based practice (*n* = 2). Nine sources described theoretical studies while the remaining 20 sources presented empirical research. Quantitative research (*n* = 11), action research (*n* = 4), qualitative research (*n* = 1), and design-based research (*n* = 1) were the research methods used in empirical sources. However, three theoretical studies did not explicitly mention their research design and were included for review.

### Themes on the Application of Scaffolding in Health Sciences Programmes

Content analysis on scaffolding application in health sciences programmes produced four themes: “Sequencing the educational activities”, “Tools and resources used for scaffolding”, “Frameworks for applying scaffolding”, and “Modelling and fading to scaffold learning”.

#### Sequencing the Educational Activities

Reviewed studies reported how health sciences programmes designed and sequenced curricula content and teaching–learning activities to enhance the scaffolding of the learning process. Dawn et al. [37] defines sequencing of educational activities as a deliberate structuring of related content, teaching–learning activities, and assessments to enhance students’ comprehension and performance of targeted competencies. Three sub-themes reflect the sequencing of educational activities, namely (1) sequencing of the content, (2) sequencing the complexity of the task, and (3) sequencing the learning environment.

##### Sequencing of the Content

Lauerer et al. [38] applied scaffolding in sequencing the content on behavioural health concepts, while Frantz and Rhoda [39] and Zook et al. [40] sequenced content related to IPE. Coombs [41] and Duff et al. [42] sequenced content on community and oral health and started with patient assessments before functional knowledge on diagnosing pathologies, planning, and implementing interventions to solve identified problems. Enquiry-based learning [43] and presentations [37, 44] were also presented as approaches to sequencing content.

##### Sequencing the Complexity of the Task

Educators meticulously structured learning tasks focusing on mastery of single or simple tasks or part of a task to complex real-life cases that demanded integration of knowledge drawn from diverse sources [45, 46]. Duff et al. [42] designed an oral health midwifery module starting with activities that aim to foster comprehension of oral cavity anatomy and physiology before mapping simulations and actual clinical practice activities that required the application and integration of knowledge on oral health pathologies. To assist nurses in learning new work-related software skills, Luo and Kalman [47] sequenced procedural steps that built on each other in facilitating students to complete tasks independently. Miller et al. [46] utilised a scaffolded sequence of writing assignments to overcome academic writing exertions. Students constructed foundational knowledge on IPE [40], behavioural health concepts [38] and community health [41] before engaging in intra- or inter-professional teams [38, 40, 41]. Frantz and Rhoda [39] designed an IPE curriculum following scholarships of teaching, application, integration, and discovery, while Coombs [41] assigned students to work on patient assessments skills before creating a care plan. Various student-centred instructional techniques, such as case studies, group projects [37], and demonstrations [48, 49], were also used in sequencing tasks.

##### Sequencing the Learning Environment

The reviewed studies showed that scaffolding in health sciences programmes was applied in various learning environments, such as the classroom, virtual space, clinical skills laboratory, the clinical setting, and the community. Modules were designed by structuring and sequencing learning to start from the either the face-to-face classroom or an online classroom, flipped classrooms [50] through simulated environments [44] and ending with learning in the clinical or community contexts [39–42]. An example was that of a multi-semester module that was designed to start with classroom learning of related IPE principles and core domains before assigning students to online IPE learning activities and communities to manage virtual or real-case problems requiring inter-professional teamwork and collaborative care [40]. Similarly, Duff et al. [42] explained how they deliberately increased learning of oral health from low to high fidelity by assigning students to engage in classroom activities before engaging in simulation laboratory activities. Concept-based simulations were deliberately structured and sequenced to alternate with theoretical classes following Benner’s novice–expert competency framework [51] to encourage nursing students to simulate simple concepts before intermediate and complex skills [52].

Clinical and physical assessment skills learning was facilitated through intertwining classroom and practical sessions [48, 49]. Students had an opportunity to apply newly acquired knowledge immediately, which aided their understanding and performance of relevant professional competencies [48, 49]. The authors reported that students were assigned to work on authentic clinical cases, presenting to class, self-evaluation, self-critique to nurture metacognition skills essential for competency development [37, 43, 53].

#### Tools and Resources Used for Scaffolding

Both paper-based and computer-based tools were used for scaffolding. Paper-based, technologic, or computer-based scaffolds offer structure and support to demonstrate relevant aspects and processes required to complete a task [54].

##### Paper-Based Tools and Resources

Paper-based tools, such as the vocabulary worksheets, self-monitoring rubrics, question prompts, self-assessments, guided notes, checklists, worked exemplars, procedure guides [37, 48–50, 53, 55], clinical reasoning scaffolds [43], peer coaching tools [56], the Graduated Descriptors Competency Tool [57], and the playbook [58], were used to support the learning of stated outcomes. The tools mainly focused on providing structural guidance on clinical practice learning, academic writing, and critical thinking and reflection [56–58]. Peinhardt and Hagler [56] demonstrate how a peer-coaching tool structures assignments to improve reliability, relevance, and evidence-based practice. Similarly, the Graduated Descriptors Competency Tool [57] maps the expected competencies of pharmacy students at every stage of their clinical development with corresponding support strategies to scaffold their development. The tool aids students to reflect on their performances and scaffolds them in identifying areas for improvement [57]. The playbook presents multiple semesters of disorienting dilemmas scaffolded to improve the learning of clinical reasoning [58]. Application of these tools resulted in the learning of physical assessment [48], clinical skills [49], clinical reasoning, and problem solving [43, 53].

##### Computer-Based Tools and Resources

Four technologic and computer-based tools were used to create virtual interactive platforms of learning. The tools and resources were the SlideTutor intelligent tutoring system [59], the SIDNIE (Scaffolded Interviews Developed by Nurses in Education) virtual platform [60], e-Support4U [61], and the CASUS electronic learning environment [62]. The SlideTutor has pedagogic models to scaffold students’ diagnostic reasoning skills. The system was automated to identify corrective actions and errors and to provide prompt hints depending on the students’ accuracy in diagnosing dermatopathology [59]. Similarly, the SIDNIE virtual platform was designed to provide adaptable scaffolding hints according to a student’s performance in interviewing a virtual patient [60, 63]. The SIDNIE and Slide tutors have built-in cognitive systems to enhance customising and adjusting feedback and support provided to students depending on the accuracy of their individual performances [59, 60]. The e-Support4U platform was structured to scaffold learning of academic writing skills through interactive e-tivities [61]. e-Support4U tools provide a rich array of supports that make learning of complex writing skills practical [61]. E-tivities enhance students’ collaboration in organising resources, content, and contributions towards completing assignments [61]. The CASUS system was designed to infuse case representation scaffolds that guide students in developing appropriate clinical reasoning skills and diagnostic pathways [62]. Additionally, Bingen et al. [50] and Lauerer et al. [38] report infusing principles of blended learning pedagogy and tools to facilitate and support learning the human physiology module and primary healthcare behavioural health concepts. Computer-based tools and virtual platforms promote the customised scaffolding of many students at one goal, a feat almost impossible when using human instructors.

#### Frameworks for Applying Scaffolding

Six studies described frameworks for applying scaffolding. Frameworks refer to detailed guides on structuring, sequencing content and learning activities, forming a support base for students to build knowledge [64]. Frameworks cited in the articles included (1) activate, plan, learn, use and show (APLUS) [44]; (2) research skills development (RSD) [65]; (3) Sawyers’ “learn, see, practice, prove, do and maintain” [66]; (4) generic consultation skills (GeCoS) and competencies [67]; (5) 6D approach — didactic, designate, distribute, design, deliver and discuss [68]; and (6) the nursing process [69] (see Table 1). These frameworks are used for structuring learning tasks [44, 65–68], for supporting the learning of clinical pathways [67, 69], for scaffolding feedback [67], and for scaffolding assessments [65, 67, 69].

#### Modelling and Fading to Scaffold Learning

Scaffolding strategies used during class engagements allowed the educator and students to change roles on multiple occasions during the learning journey. Initially, the educator took an active role by conducting introductory sessions on the fundamentals of physical examination [48], principles of literature evaluation and text organisation [37], and foundational content related to a clinical skill [49]. The educator’s role then progressed to include modelling problem-solving processes [43, 48, 53], demonstrating physical assessment, clinical skills, and information retrieval processes [37, 48, 49, 55]. The educator’s roles gradually lessened in later stages through fading. Both physical and virtual support faded as students individually or collaboratively took over the responsibility to complete complex tasks, such as a critical appraisal of clinical literature [37], managing ill-structured problems [53], conducting a physical assessment [48], and performing relevant clinical skills [49].

## Discussion

The purpose of this integrative review was to describe the application of scaffolding in health sciences programmes. High-income countries such as the USA and Australia dominated the number of sources included in this review, possibly due to limited uptake of student-centred education approaches in low-income settings [70, 71]. Student-centred approaches necessitate the application of scaffolding. As expected, nursing, midwifery and medicine reported the most on scaffolding in their programmes because they generally have more students than other disciplines in health sciences [72, 73]. This review integrated data from theoretical and empirical studies whose quality was generally good. Four themes reflected the application of scaffolding in health sciences programmes.

Sequencing of the educational content, learning tasks and learning environments followed increasing complexity to enhance students’ progress in competency development. Scaffolding has been described as both a design of content and a process of delivering such content to support meaningful learning [41]. Educators arranged content to begin with foundational sciences forming a basis for decision-making in clinical practice [50, 59, 68], a view supported by the work of Dickinson et al. [74] as well as Grande [75]. The sequencing of the content and learning tasks promoted learning built on prior knowledge [38, 47]. Learning tasks were scaffolded by focusing on single, small successive chunks of foundational concepts before complex situations that demand knowledge integration [40, 45, 69]. Similar programme design principles related to task scaffolding were described as requirements for enhancing the learning of complex skills, such as scaffolding metacognition, diagnostic reasoning, clinical reasoning, psychomotor skills, inter-professional practice, academic writing, and designing treatment plans [76–78]. In scaffolding learning platforms, students were first exposed to theory in classrooms, then moved to applying the theory in simulation before work-integrated learning [40, 42, 45]. This approach to sequencing learning platforms promoted building cognitive bridges between theoretical knowledge and experiential learning among students [79]. Sequencing content, tasks and learning platforms allowed students to engage with learning material and experiences, promoting knowledge internalisation and automatisation.

Various scaffolding tools were used during face-to-face and virtual engagement with learning materials, such as the playbook [58] and e-tivities [61]. These student-centred tools provided “just-in-time” scaffolds, such as prompts, hints, examples, and checklists, to ease students’ cognitive processing and reduce their cognitive load [45]. According to the saw-tooth model, presentation of supportive information or “just-in-time” scaffolds increase students’ chances of completing the required task [80]. Computer-based tools, such as virtual simulations, software systems, digital tools, and other technological advancements, were used to provide optimum learning support [56, 57, 59–62]. Although there is a risk of “blanket scaffolding” [54], tools offer structure and multimodal scaffolding support for students to complete a learning task with ease [4, 54, 81]. The recent growth in technology inadvertently supported the adoption of technological scaffolding innovations in higher education [4, 25, 82, 83]. Hence, exploitation of opportunities presented by improvements in technology is a remedy for programmes with large student cohorts. The design of computer-mediated systems with cognitive and metacognitive functions assisted in providing dynamic scaffolding for learning essential clinical skills, such as diagnostic reasoning and communication, which are usually learnt during patient interactions [59, 62]. The adaptive capacity, dynamism, contingency, and fading witnessed in computer systems used in health sciences programmes improved the limitations of cultural tools described in the work of Malik [84].

Frameworks were applied to structure the process of learning and the design of learning activities [85]. The authors applied frameworks in structuring learning activities in progressive steps built on one another [66–68], and students learnt through applying clinical pathways in practice [65, 69]. Frameworks are essential in enhancing transferability and standardisation of scaffolding interventions across platforms and contexts allowing for vertical scaffolding in programmes [65, 86].

In the initial stages of learning a concept, educators play an active role including modelling of expected outcomes and facilitation of learning. The educator’s engagement faded as students became independent as evidenced by knowledge internalisation and automatisation. Van Merriënboer and Sweller [80] argue that the processes of modelling and fading need to be repeated with each subsequent learning outcome to build competence.

### Study Limitations

The search string was in English, which excluded studies published in other languages. Therefore, the outcome of this review is biased towards Anglophone populations. The use of a single university database interface may have influenced the literature search outputs limiting the representativeness of this review. The inconsistency in the definition and application of the concept of scaffolding in the literature may have influenced the inclusion and exclusion of studies for review.

### Suggestions for Further Research

Studies included in this review reported the application of scaffolding mostly in single modules or learning platforms. Holistic programme-wide scaffolding strategies should be developed and implemented within student-centred health sciences programmes. Future research could investigate the consistent use of frameworks and models related to scaffolding across all health sciences programmes and in different settings and contexts.

## Conclusion

The complexity of health sciences programmes justifies scaffolding to support students in becoming competent health professionals. This integrative review summarises the application of scaffolding in health sciences programmes. The sequencing of educational activities, tools or resources used in scaffolding, frameworks for applying scaffolding, and instructional strategies such as modelling and fading represented the application of scaffolding in health sciences programmes. The reviewed studies demonstrated the deliberate design of educational activities aligned with the students’ zone of proximal development. Such activities supported students’ transition from low fidelity and low complexity with high support to become independent tasks performers in complex environments with faded or no support from the educator. Educators should include scaffolding during instructional design and teaching–learning interactions. Scaffolding in health sciences should address various learning platforms, including simulation, clinical learning platforms and also goes beyond face-to-face interactions. Therefore, health sciences programmes need multifaceted, systematic, and programmatic approaches to scaffolding that begin with meticulous planning, design, and sequencing of curricula to build up necessary learning experiences for students.

## Data Availability

The data generated or analysed during this review is included in this manuscript. Raw data extract data from selected sources and presented in author-generated data charting form is available on request.
